# Long non‐coding RNA TUG1 promotes airway remodelling by suppressing the miR‐145‐5p/DUSP6 axis in cigarette smoke‐induced COPD

**DOI:** 10.1111/jcmm.14389

**Published:** 2019-09-26

**Authors:** Wenchao Gu, Yaping Yuan, Linxuan Wang, Hua Yang, Shanshan Li, Zhijun Tang, Qiang Li

**Affiliations:** ^1^ Department of Respiratory Medicine, Changhai Hospital Second Military Medical University Shanghai China; ^2^ Department of Respiratory Medicine Pudong New Area Peoples’ Hospital Affiliated to Shanghai Health University Shanghai China

**Keywords:** airway inflammation, airway remodelling, COPD, DUSP6, miR‐145‐5p, TUG1

## Abstract

Chronic obstructive pulmonary disease (COPD) is a progressive lung disease that is primarily caused by cigarette smoke (CS)‐induced chronic inflammation. In this study, we investigated the function and mechanism of action of the long non‐coding RNA (lncRNA) taurine‐up‐regulated gene 1 (TUG1) in CS‐induced COPD. We found that the expression of TUG1 was significantly higher in the sputum cells and lung tissues of patients with COPD as compared to that in non‐smokers, and negatively correlated with the percentage of predicted forced expiratory volume in 1 second. In addition, up‐regulation of TUG1 was observed in CS‐exposed mice, and knockdown of TUG1 attenuated inflammation and airway remodelling in a mouse model. Moreover, TUG1 expression was higher in CS extract (CSE)‐treated human bronchial epithelial cells and lung fibroblasts, whereas inhibition of TUG1 reversed CSE‐induced inflammation and collagen deposition in vitro. Mechanistically, TUG1 promoted the expression of dual‐specificity phosphatase 6 (DUSP6) by sponging miR‐145‐5p. DUSP6 overexpression reversed TUG1 knockdown‐mediated inhibition of inflammation and airway remodelling. These findings suggested an important role of TUG1 in the pathological alterations associated with CS‐mediated airway remodelling in COPD. Thus, TUG1 may be a promising therapeutic target in CS‐induced airway inflammation and fibroblast activation.

## 1 INTRODUCTION

Chronic obstructive pulmonary disease (COPD) is a progressive degenerative lung disease and currently the fourth leading cause of death worldwide.^1^ Cigarette smoke (CS) is the most important cause of COPD, and smoking cessation early in the course of the disease can slow the rate at which lung function is lost. CS is the major aetiological factor in the development of COPD. Because the airway epithelium is the primary target of inhaled harmful particles, abnormal tissue repair has become the primary research focus for elucidating the process of airway remodelling.^2^ Functions of lung fibroblasts are altered in COPD in multiple ways, including extracellular mediator production, inflammatory cytokine production and cellular proliferation.^3^ Airway remodelling is a critical feature of COPD and is characterized by aberrant repair of the epithelium and accumulation of fibroblasts.^4^

Long non‐coding RNAs (lncRNAs) are defined as non‐coding RNAs that have a length greater than 200 nucleotides. They play crucial roles in the regulation of gene expression.^5^ The rapid development of RNA genomics has highlighted the involvement of various lncRNAs in COPD, such as LISPR1 and ANRIL.^6^, ^7^ The lncRNA taurine‐up‐regulated gene 1 (TUG1), a ~7.1 kb highly conserved lncRNA, was originally identified in taurine‐treated retinal cells. TUG1 has been reported to play critical roles in the progression of many human diseases, such as cancer, aortic valve calcification and pre‐eclampsia.^8^, ^9^, ^10^ Indeed, TUG1 can regulate gene expression via diverse mechanisms during various biological processes, including inflammatory response. Overexpression of TUG1 has been shown to protect the mouse liver against cold‐induced injury by inhibiting apoptosis and inflammation,^11^ whereas TUG1 knockdown inhibits hyperlipidaemia, decreases inflammatory response and alleviates atherosclerotic lesions.^12^ COPD is primarily caused by CS‐induced chronic inflammation. Nevertheless, the biological functions and mechanism of action of TUG1 in CS‐induced COPD remain unclear.

In this study, we showed that the expression of TUG1 is up‐regulated in the sputum cells and lung tissues of patients with COPD compared to that in non‐smokers, and negatively correlated with the percentage of predicted forced expiratory volume in 1 second (FEV1%). Knockdown of TUG1 reversed CS‐induced airway remodelling by inhibiting airway inflammation and airway remodelling in vitro and in vivo. Moreover, we found that TUG1 promoted the expression of dual‐specificity phosphatase 6 (DUSP6) through sponging miR‐145‐5p, and DUSP6 reversed the inhibitory effect of TUG1 knockdown on airway remodelling. These results suggested that TUG1 is a promising therapeutic target in CS‐induced airway inflammation and fibroblast activation.

## 2 MATERIALS AND METHODS

### 2.1 Study population for human lung tissue sampling

Lung resection samples were obtained from five never‐smokers (non‐smokers), five current smokers without COPD (smokers) and 10 current smokers with COPD (group COPD). The study protocols were approved by the Institutional Ethics Committee of Second Military Medical University. Written informed consent was obtained from all the participants. Lung samples were frozen in liquid nitrogen after collection and stored at −80°C until use for RNA isolation.

### 2.2 Study population for human sputum sampling

Healthy individuals (15 non‐smokers and 20 smokers) and 30 smokers with COPD were enrolled in the induced sputum experiment. There was no overlap between the patient groups for lung tissue and sputum analysis. Each patient inhaled a 3% saline atomized solution and then spat out saliva, took two deep inspirations of saline, and coughed sputum into a separate cup. Dithiothreitol was added to the sputum samples, which were then mixed vigorously on a plate shaker to solubilize the mucus.

### 2.3 Plasmid construction, lentivirus production, and cell transduction

miR‐145‐5p mimic and its negative control mimic (miR‐NC) and miR‐145‐5p inhibitor and its negative control inhibitor were purchased from RiboBio (Guangzhou, China). The sequences as follows: miR‐145‐5p mimic, 5′‐GUCCAGUUUUCCCAGGAAUCCCU‐3′ and 5′‐GGAUUCCUGGGAAAACUGGACUU‐3′; Negative control, 5′‐UUCUCCGAACGUGUCACGUTT‐3′ and 5′‐ACGUGACACGUUCGGAGAATT‐3′; miR‐145‐5p inhibitor, 5′‐AGGGAUUCCUGGGAAAACUGGAC‐3′; scrambled control, 5′‐CAGUACUUUUGUGUAGUACAA‐3′. The coding region of the DUSP6 mRNA was cloned into the pcDNA3.1(+) vector. The lentiviral vector expressing short hairpin RNA (shRNA) targeting TUG1 was designed and constructed by GenPharma (Shanghai, China). All cell transfection procedures were performed with Lipofectamine 2000 (Life Technologies Inc, Carlsbad, CA, USA).

### 2.4 Animal exposure procedures

Animal experiments were conducted in accordance with the guidelines of the Institutional Animal Use and Care Committee at Second Military Medical University. C57BL/6 mice (6‐8 weeks old) were exposed to the smoke of five cigarettes (Jiao Zi, Chengdu Cigarette Factory, Chengdu, China; without filter) via a TE‐10 smoking machine as described previously.^13^ The mice were exposed to CS four times each day with 30‐minute smoke‐free intervals, 5 days/week for up to 15 weeks. The TUG1 shRNA‐expressing lentivirus or empty lentivirus was intranasally injected into the mice once every 2 weeks. The control groups were exposed to room air. The mice were killed at 24 hours after the last exposure.

### 2.5 Collection of bronchoalveolar lavage fluid

Lungs were lavaged with a cannula inserted into the trachea and instilled with phosphate‐buffered saline (PBS) after exsanguination. Bronchoalveolar lavage fluid (BALF) cells stained with the Wright‐Giemsa dye were identified under a light microscope. The total numbers of leucocytes and eosinophils were counted under the microscope in a blinded manner.

### 2.6 Quantitative reverse transcriptase polymerase chain reaction

Total RNA extraction from the lung tissues and cells was performed with the TRIzol Reagent Kit (Life Technologies) and converted into cDNA using reverse transcriptase (Life Technologies). Quantitative reverse transcriptase polymerase chain reaction (qRT‐PCR) was carried out on an ABI StepOnePlus Real‐Time PCR System. The relative expression levels were calculated by the 2^–ΔΔCt^ method, and data were normalized to those for U6 small nucleolar RNA or β‐actin as endogenous controls. The primer sequences include: TUG1, 5′‐CTGAAGAAAGGCAATCCATC‐3′ and 5′‐GTAGGCTACTACAGGTCATTTG‐3′; miR‐145‐5p, 5′‐CAGTCTTGTCCAGTTTTCCCAG‐3′ and 5′‐TATGCTTGTTCTCGTCTCTGTGTC‐3′; DUSP6, 5′‐CAGTGGTGCTCTACGACGAG‐3′ and 5′‐GCAATGCAGGGAGAACTCGGC‐3′; IL‐6, 5′‐GACCCAACCACAAATGCCAG‐3′ and 5′‐GCTGCGCAGAATGAGATGAG‐3′; TGF‐β1, 5′‐CAACTCCGGTGACATCAAAA‐3′ and 5′‐ACGTGGAGCTGTACCAGAAA‐3′; collagen I, 5′‐GAGAGCATGACCGATGGATT‐3′ and 5′‐ CCTTCTTGAGGTTGCCAGTC‐3′; α‐SMA, 5′‐GAAGAAGAGGACAGCACT‐3′ and 5′‐TCCCATTCCCACCATCAC‐3′; β‐actin, 5′‐CTGGGACGACATGGAGAAAA‐3′ and 5′‐AAGGAAGGCTGGAAGAGTGC‐3′; U6, 5′‐TGGAACGCTTCACGAATTTGCG‐3′ and 5′‐ AGACTGCCGCCTGGTAGTTGT‐3′.

### 2.7 Western blot analysis

Total protein samples were prepared using RIPA buffer, separated by SDS‐PAGE, transferred to a nitrocellulose membrane, and then incubated with an anti‐DUSP6 antibody (Cell Signaling Technology, Beverly, MA, USA), anti‐α‐SMA antibody (Proteintech, Wuhan, China), or anti‐collagen I antibody (Proteintech) overnight at 4°C. After washing with TBST, membranes were incubated with secondary antibodies for 1 hour. The protein bands were visualized using an enhanced chemiluminescence system. Equal loading of samples was verified by immunoblotting for β‐actin. Band intensities were quantified using Image Gauge software.

### 2.8 Lung histology

The lung tissue samples of mice were fixed in 10% formalin, embedded in paraffin and then sliced into 3‐4 μm sections. The lung sections were stained with hematoxylin and eosin. They were subjected to immunostaining procedures and Masson staining as previously described.^14^ The area of peribronchial Masson trichrome staining (blue colour) was visualized and quantified. The α‐SMA protein was identified in the paraffin‐embedded sections of the lung tissue by immunohistochemical staining with an α‐SMA antibody (Proteintech). The sections were examined under a light microscope and the data were quantified using Image Pro 6.0 software. Results are expressed as the area of α‐SMA staining per micrometer length of bronchioles’ basement membrane of 150‐200 μm in internal diameter.

### 2.9 Immunofluorescence

Cells were fixed in ice‐cold methanol, washed with PBS, blocked with 3% bovine serum albumin and incubated with primary antibodies for 1 hour. The cells were then immunostained for α‐SMA and collagen I (Proteintech), and then viewed under a fluorescence microscope.

### 2.10 Cell culture

Human bronchial epithelial (HBE) cells were purchased from American Type Culture Collection and maintained in RPMI 1640 supplemented with 10% foetal bovine serum (FBS), 50 U/mL penicillin, and 50 U/mL streptomycin in an incubator at 37°C and 5% of CO_2_. Primary fibroblasts were isolated as described previously.^15^ Proximal lung tissue containing small airways was minced into 1‐2 mm pieces and centrifuged at 300 *g*. The supernatant was aspirated, and the tissue pellet was re‐suspended and plated onto tissue culture grade plastic flasks in Dulbecco’s modified Eagle’s medium (DMEM) containing 10% FBS and 2% antibiotics. The epithelial layer was removed, followed by blunt dissection of airway smooth‐muscle bundles out of the section. The remaining tissue was then minced into 1‐2 mm fragments and added to tissue culture flasks. Identification of fibroblasts was based on the expression of vimentin.

### 2.11 CS extract preparation

Cigarette smoke extract (CSE) preparation was carried out as described previously.^16^ Smoke from 10 cigarettes (Chengdu Cigarette Factory) was bubbled through DMEM, which was regarded as 100% CSE, and then frozen and stored at −80°C until further use. This CSE was diluted with medium to a final concentration of 5% for this study.

### 2.12 Luciferase reporter assay

Plasmid pmirGLO‐TUG1 wild‐type (wt) or pmirGLO‐TUG1 mutant (mut) (relevant binding sites in miR‐145‐5p) was co‐transfected with miR‐145‐5p mimics or miR‐NC into HEK293T cells using a Lipofectamine‐mediated gene transfer. DUSP6‐wt/mut 3UTR were constructed and transfected into HEK293T cells along with miR‐145‐5p mimic/miR‐NC. Luciferase activity was detected using the Dual‐Luciferase Reporter Assay System (Promega, Madison, WI, USA) according to the manufacturer’s instructions.

### 2.13 Statistical analysis

Statistical analyses were performed with GraphPad Prism version 5.0 (GraphPad Software, San Diego, CA, USA), and data are expressed as the mean ± SD. Comparisons between two groups were performed by Student’s *t* test, whereas comparisons between multiple groups were performed by one‐way ANOVA. The analysis of correlation between factors was performed by Spearman’s correlation coefficient rank test. Statistical significance was assumed at *P* < 0.05.

## 3 RESULTS

### 3.1 Abnormal expression of TUG1 in COPD

**Figure 1:**
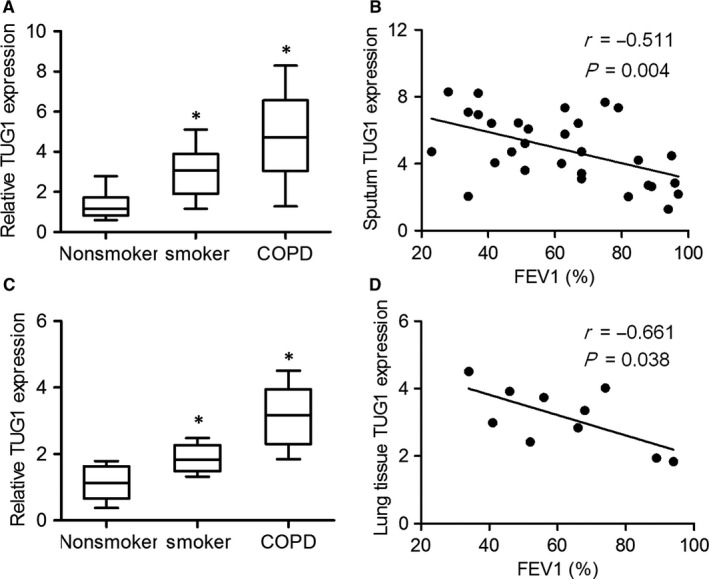
TUG1 expression was higher in patients with COPD. A, Sputum cells were obtained from non‐smokers (n = 15), smokers (n = 20) and patients with COPD (n = 30). The expression of TUG1 in sputum cells was measured by qRT‐PCR. B, Spearman’s correlation analysis between TUG1 expression and FEV1%. C, Lung tissue samples were obtained from non‐smokers (n = 5), smokers (n = 5) and patients with COPD (n = 10). The expression of TUG1 in lung tissue samples was measured by qRT‐PCR. D, Spearman’s correlation analysis between TUG1 expression and FEV1%. *P < 0.05 as compared to non‐smoker group

To determine whether TUG1 expression was associated with COPD and identify its possible correlation with airway remodelling, we performed qRT‐PCR. As shown in Figure 1A and 1, TUG1 expression in the sputum cells was higher in patients with COPD and in smokers without COPD as compared to that in non‐smokers, and negatively correlated with the FEV1% value. Consistent with the expression of TUG1 in sputum cells, TUG1 was also up‐regulated in the lung tissues of patients with COPD, and its expression in the lung tissues of patients with COPD was significantly correlated with FEV1% (Figure 1C and 1). These results indicated that TUG1 levels increased significantly as COPD progressed.

### 3.2 Knockdown of TUG1 protected against airway remodelling after CS exposure in vivo

**Figure 2:**
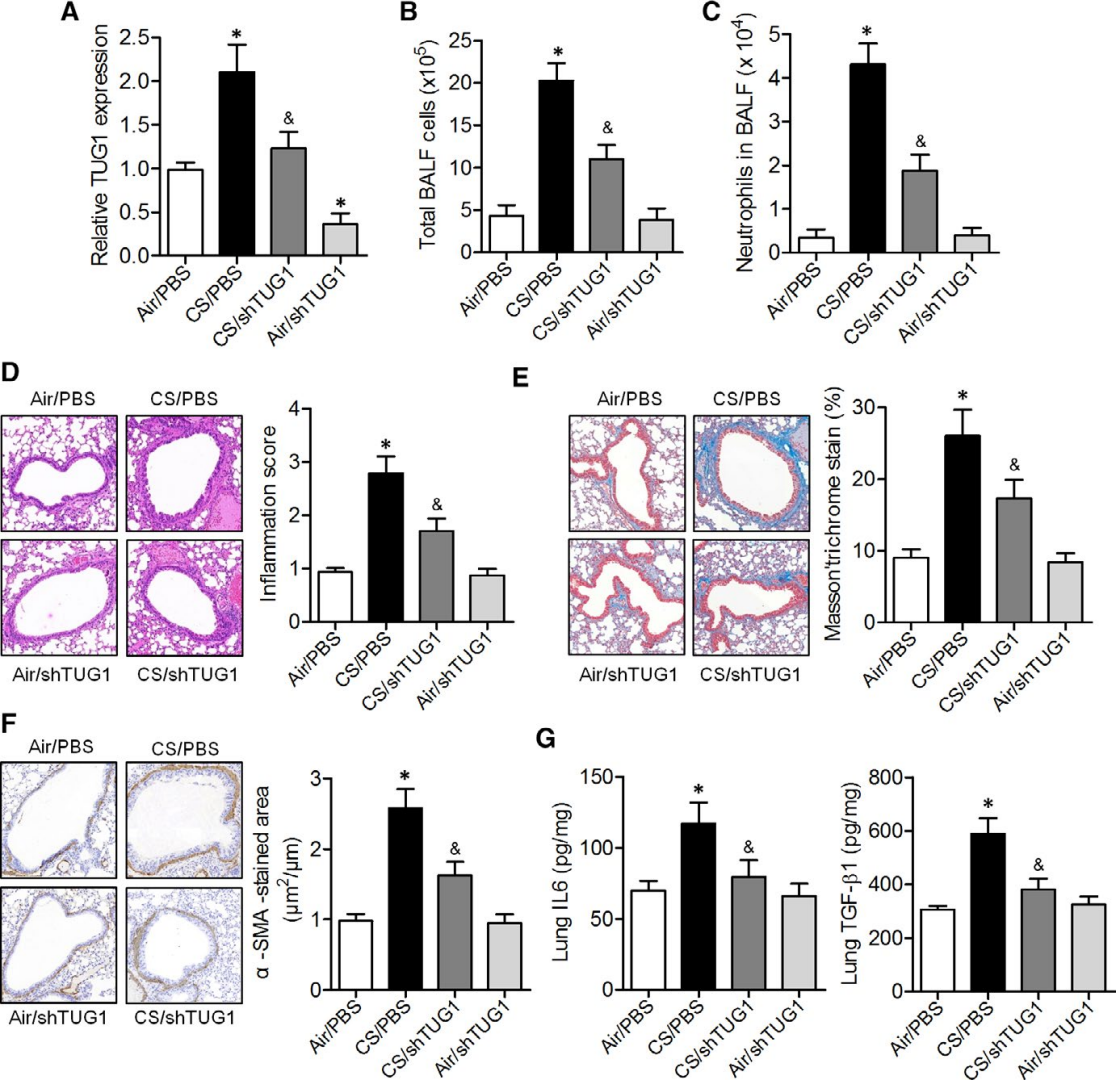
Knockdown of TUG1 inhibited CS‐induced airway remodelling and airway inflammation in vivo. A, TUG1 expression was assessed by qRT‐PCR in the lung tissues of air‐ or CS‐exposed mice transduced or not transduced with the TUG1 knockdown construct. B‐D, Counting of total bronchoalveolar lavage fluid (BALF) cells and neutrophils, and histological analysis of the lung sections were performed by haematoxylin and eosin staining to visualize inflammatory cell recruitment. E and F, Representative Masson and α‐SMA staining of lung sections from air‐ or CS‐exposed mice transduced or not transduced with the TUG1 knockdown construct. G, The expression of the inflammatory cytokines IL‐6 and TGF‐β1 in the lung tissues was quantified by ELISA. *P < 0.05 versus Air/PBS group; ^&^ *P* < 0.05 as compared to CS/PBS group

To determine the potential effect of TUG1 on the pathogenesis of airway remodelling, we intranasally administered a TUG1 knockdown lentivirus to mice exposed to CS for 15 weeks. TUG1 expression was significantly higher in the lung tissues of CS‐exposed mice as compared to that in air‐exposed mice, whereas TUG1 expression was lower in CS‐exposed mice transduced with the TUG1 knockdown lentivirus (Figure 2A). The total numbers of cells and neutrophils in BALF were significantly greater in CS‐exposed mice, and were reduced by TUG1 knockdown (Figure 2B and 2). Haematoxylin and eosin staining revealed an increase in the levels of peribronchial infiltrates of inflammatory cells in CS‐exposed mice, and these levels were decreased by TUG1 knockdown (Figure 2D). The parameters of airway remodelling, such as peribronchial collagen deposition and smooth‐muscle mass, were obviously lower in CS‐exposed mice transduced with the TUG1 knockdown lentivirus as compared to those in CS‐exposed mice treated with PBS (Figure 2E). In addition, we noted that the levels of the pro‐inflammatory factors, interleukin (IL)‐6 and transforming growth factor (TGF)‐β1 were significantly higher in the lung tissues of CS‐exposed mice and lower in CS‐exposed mice transduced with the TUG1 knockdown lentivirus (Figure 2F and G). These results suggested that knockdown of TUG1 inhibited inflammatory cell influx and airway remodelling in the lungs of mice in response to CS exposure.

### 3.3 Knockdown of TUG1 reduced CSE‐triggered inflammation and airway remodelling in HBE cells and lung fibroblasts in vitro

**Figure 3:**
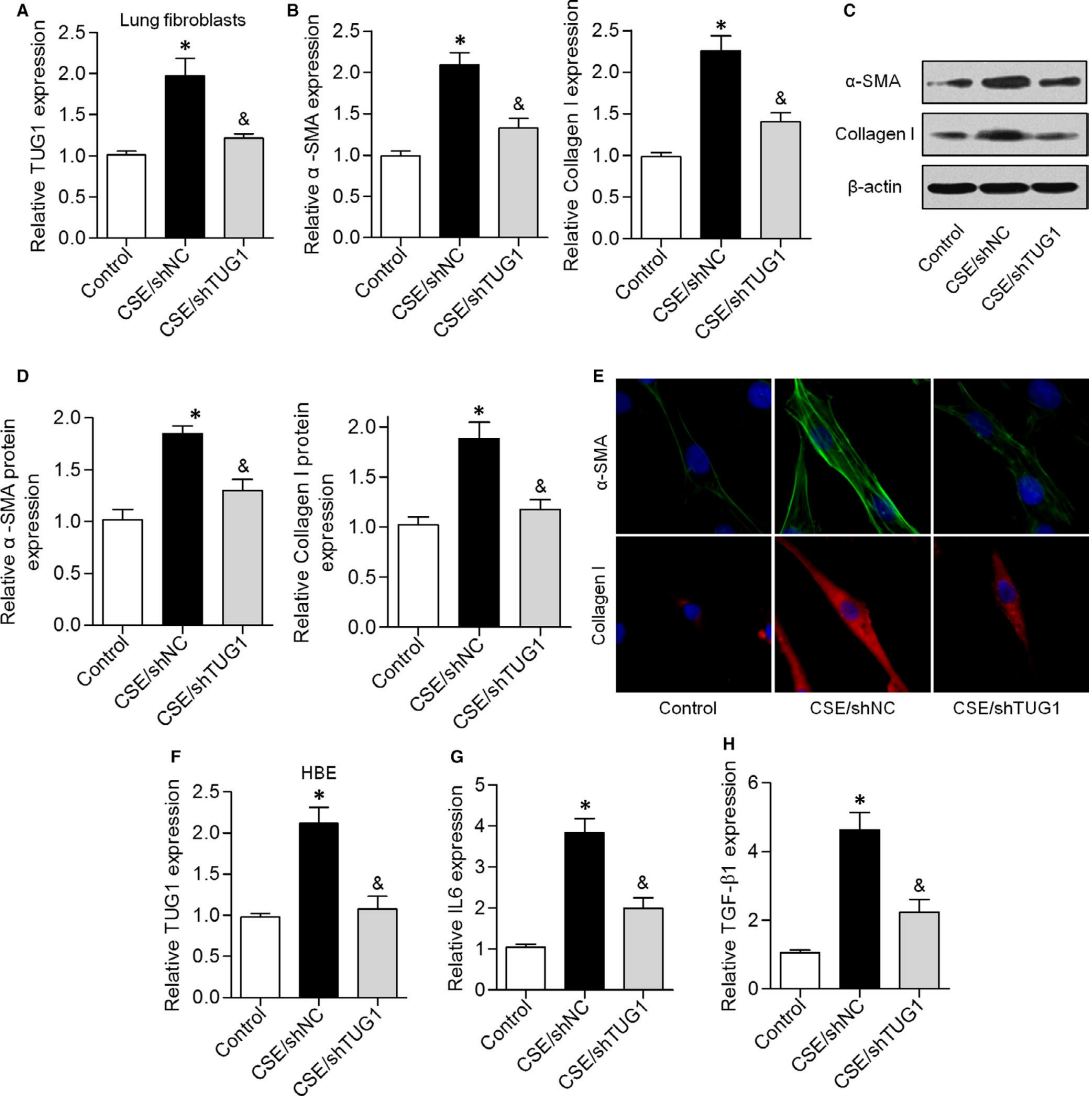
Knockdown of TUG1 inhibited CSE‐induced airway remodelling and airway inflammation in vitro. A, The expression of TUG1 was analysed in CSE‐treated parenchymal fibroblasts. B‐D, The mRNA and protein expression levels of α‐SMA and collagen I were determined by qRT‐PCR and Western blot analysis, respectively, in these groups. E, α‐SMA and collagen I expression was detected by immunofluorescent staining. F, Expression of TUG1 was analysed in HBE cells treated with 5% CSE for 48 h and transfected with shTUG1. G and H, Expression levels of IL‐6 and TGF‐β1 were assessed by qRT‐PCR. *P < 0.05 as compared to control group; ^&^ *P* < 0.05 as compared to CSE/shNC group

Fibroblasts function as a major source of airway fibrosis and extracellular‐matrix protein deposition, which lead to peripheral airway narrowing in COPD.^17^, ^18^ We showed that TUG1 expression was higher in CSE‐treated lung fibroblasts and lower in CSE/shTUG1‐treated lung fibroblasts (Figure 3A). The mRNA and protein expression levels of α‐SMA and collagen I were significantly higher in CSE‐treated lung fibroblasts, but these levels were reduced by shTUG1 treatment (Figure 3B‐D). Consistent with the above results, immunofluorescent staining indicated that knockdown of TUG1 dramatically suppressed the increase in α‐SMA and collagen I levels induced by CSE exposure (Figure 3E). We next investigated the effects of CSE exposure on HBE cells. CSE exposure significantly up‐regulated TUG1 expression in HBE cells, and this effect was attenuated by TUG1 knockdown (Figure 3F). qRT‐PCR results indicated that CSE exposure induced an increase in IL‐6 and TGF‐β1 levels, while knockdown of TUG1 expression attenuated this effect (Figure 3G and H). These results suggested that knockdown of TUG1 reduced CSE‐triggered inflammation and airway remodelling in vitro*.*

### 3.4 TUG1 positively regulated DUSP6 expression via sponging miR‐145‐5p

**Figure 4:**
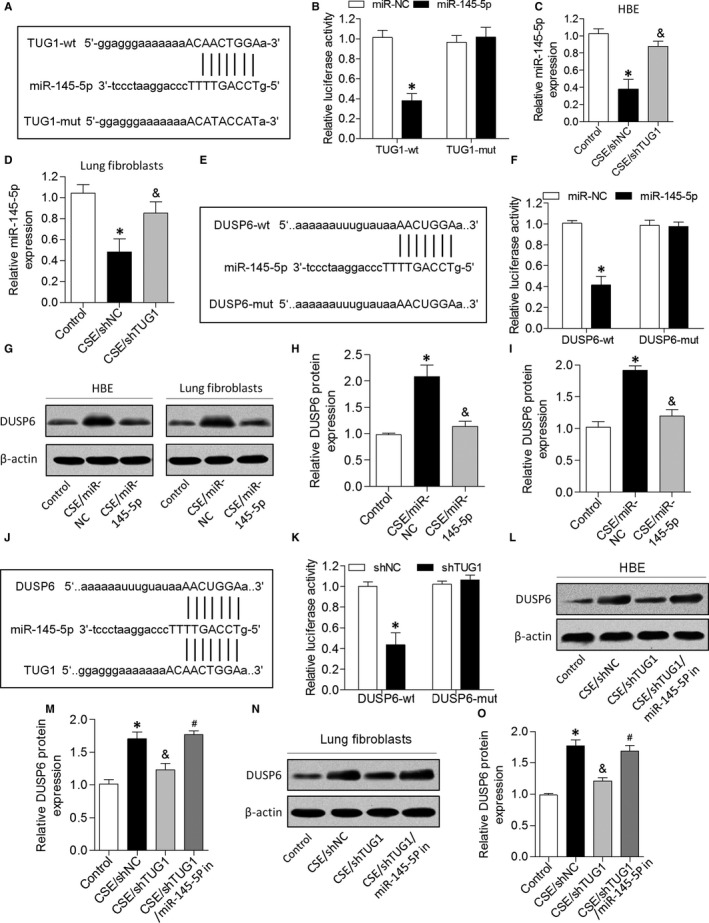
TUG1 positively regulated DUSP6 expression by sponging miR‐145‐5p. A, The putative miR‐145‐5p–binding sequence of *TUG1* mRNA. B, Luciferase activity was measured. C and D, Expression of miR‐145‐5p was analysed in CSE‐treated HBE cells and lung fibroblasts transfected with the TUG1 knockdown construct. E, wt or mut miR‐145‐5p target sequences of the 3′UTR from *DUSP6*. F, Luciferase activity was measured. G‐I, DUSP6 expression was detected by Western blotting in CSE‐treated HBE cells and lung fibroblasts transfected with miR‐145‐5p mimic. J, The putative miR‐145‐5p–binding sequence of *TUG1* and *DUSP6*. K, Luciferase activity was measured. L‐O, The protein expression of DUSP6 was measured in different groups. *P < 0.05 as compared to control group; ^&^ *P* < 0.05 as compared to CSE/shNC group or CSE/miR‐NC group; ^#^ *P* < 0.05 as compared to CSE/shTUG1 group

To examine the mechanisms by which TUG1 exerted its effects on the pathogenesis of airway remodelling, we predicted miRNA target sites using an online bioinformatic database and identified miR‐145‐5p as an lncRNA with relevant binding sites in the *TUG1* mRNA (Figure 4A). We constructed luciferase reporter vectors that contained the wild‐type (wt) or mutated (mut) binding sequences for miR‐145‐5p in *TUG1*, and luciferase reporter assay results showed that luciferase activity was suppressed in TUG1‐wt cells but was not affected in TUG1‐mut cells, suggesting that miR‐145‐5p is a *TUG1*‐targeting miRNA (Figure 4B). Further, we demonstrated that miR‐145‐5p expression was down‐regulated in CSE‐treated HBE cells and lung fibroblasts, but both parameters were increased by TUG1 knockdown (Figure 4C and 4). Furthermore, we found that miR‐145‐5p interacted with the 3UTR of *DUSP6* mRNA (Figure 4E). Dual‐luciferase reporter assay revealed that co‐transfection with DUSP6‐wt and miR‐145‐5p mimic significantly inhibited luciferase activity, whereas co‐transfection with DUSP6‐mut and miR‐145‐5p mimic failed to affect luciferase activity (Figure 4F). CSE exposure caused an increase in DUSP6 expression in HBE cells and lung fibroblasts, but this increase was attenuated by miR‐145‐5p (Figure 4G‐I). Next, bioinformatics analysis predicted that *TUG1* and *DUSP6* mRNAs contain the same binding site for miR‐145‐5p, and TUG1 knockdown decreased the luciferase activity of cells transfected with DUSP6‐wt (Figure 4J and K). In addition, TUG1 knockdown significantly suppressed the expression of *DUSP6* in HBE cells and lung fibroblasts treated with CSE, and this effect was reversed by miR‐145‐5p inhibition (Figure 4L‐O). These results indicated that TUG1 positively regulated DUSP6 expression by sponging miR‐145‐5p in HBE cells and lung fibroblasts.

### 3.5 Overexpression of DUSP6 reversed the effects of TUG1 knockdown in HBE cells and lung fibroblasts

**Figure 5:**
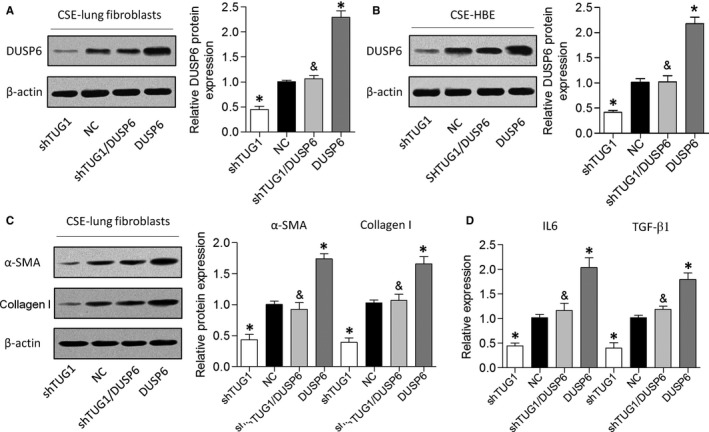
Overexpression of DUSP6 reverses the inhibition of airway inflammation and fibrosis mediated by HBE cells and lung fibroblasts after TUG1 knockdown. A and B, Expression of DUSP6 was determined in CSE‐treated HBE cells and lung fibroblasts transfected with shTUG1 or DUSP6 alone or co‐transfected with shTUG1 and DUSP6. C, Protein expression levels of α‐SMA and collagen I were measured in lung fibroblasts. D, IL‐6 and TGF‐β1 levels were measured in HBE cells. *P < 0.05 as compared to NC group; ^&^ *P* < 0.05 as compared to CSE/shTUG1 group

Finally, we performed gain‐of‐function assays by introducing a DUSP6 overexpression vector into HBE cells and lung fibroblasts with TUG1 knockdown (Figure 5A and 5). As described above, TUG1 knockdown suppressed α‐SMA and collagen I expression levels in CSE‐treated cells, whereas DUSP6 overexpression abrogated this effect (Figure 5C). Furthermore, DUSP6 overexpression attenuated the suppressive effect of TUG1 knockdown on the expression of inflammatory cytokines in HBE cells treated with CSE (Figure 5D). These results revealed that TUG1 knockdown might have a protective effect on airway remodelling in a DUSP6‐dependent manner.

## 4 DISCUSSION

Aberrant expression of lncRNAs has been associated with several pulmonary disorders, suggesting the involvement of lncRNAs in the pathogenesis of these diseases. As a relatively well‐studied lncRNA, TUG1 is deeply involved in the progression of many human diseases. It has been demonstrated that TUG1 expression is markedly higher in COPD‐affected lung tissues compared to that in non‐COPD lung tissues.^19^ Nonetheless, no further studies on the expression or function of TUG1 in COPD have been published. In this study, we verified increased TUG1 expression in the sputum cells and lung tissue samples of patients with COPD, and its negative correlation with FEV1%. Furthermore, we observed that the knockdown of TUG1 reversed CS‐induced airway inflammation and airway remodelling in vitro and in vivo.

Accumulating evidence has indicated that lncRNA acts as a competing endogenous RNA or miRNA sponge, thereby modulating a variety of cellular biological activities.^20^ For example, the lncRNA CCAT1 promoted gall bladder cancer progression by acting as miR‐218‐5p sponge and increasing the expression of its target gene *Bmi1*.^21^ The lncRNA DANCR competitively binds to miR‐335‐5p and miR‐1972 to regulate the expression of ROCK1, thereby promoting the malignant behaviour of osteosarcoma.^22^ TUG1 is also involved in many diseases by virtue of acting as a competing endogenous RNA or miRNA sponge. For instance, TUG1 promoted KIAA1199 expression via miR‐600 to accelerate cell metastasis and epithelial‐mesenchymal transition in colorectal cancer,^23^ while knockdown of TUG1 ameliorated atherosclerosis via up‐regulation of the miR‐133a target gene *FGF1*.^12^ Understanding the precise molecular mechanism by which lncRNAs function would facilitate the development of lncRNA‐directed diagnostics and therapeutics for COPD. Using the miRanda algorithm, we found that TUG1 binds to miR‐145‐5p. It was shown that the expression of miR‐145‐5p significantly diminished in response to a CS condensate and negatively regulated proinflammatory cytokine release from airway smooth‐muscle cells in COPD by targeting SMAD3.^24^, ^25^ In the present study, we found that CSE treatment down‐regulated miR‐145‐5p in HBE cells and lung fibroblasts, and this effect was reversed by TUG1 knockdown. Next, we showed that miR‐145‐5p directly interacted with the 3′UTR of *DUSP6* mRNA.

DUSP6, also known as MKP3 or PYST1, belongs to the family of mitogen‐activated protein kinase (MAPK) phosphatases that act as feedback regulators of MAPK cascades. It has been reported that DUSP6 functions as a key mediator of various physiological processes, especially inflammatory responses. For example, enhancement of macrophage inflammatory responses by CCL2 correlated with down‐regulation of the ERK1/2 phosphatase DUSP6.^26^ Hsu et al revealed that manipulation of DUSP6 holds great potential for the treatment of acute inflammatory diseases.^27^ Missinato et al reported that DUSP6 attenuated Ras/MAPK signalling during regeneration and that suppression of DUSP6 expression could enhance cardiac repair.^28^ In our study, CSE exposure up‐regulated DUSP6 in HBE cells and lung fibroblasts, and this change was reversed by miR‐145‐5p. Further experiments suggested that TUG1 knockdown suppressed α‐SMA and collagen I expression in CSE‐treated cells, whereas DUSP6 overexpression abrogated this effect. Additionally, DUSP6 overexpression alleviated the suppressive effect of TUG1 knockdown on the expression of inflammatory cytokines in CSE‐treated HBE cells.

In conclusion, we demonstrated that TUG1 knockdown inhibited inflammation and airway remodelling in COPD through in vitro and in vivo assays. Importantly, we found that TUG1 promoted the expression of DUSP6 by sponging miR‐145‐5p. Understanding the underlying molecular mechanism would facilitate the development of lncRNA‐directed diagnostics and therapeutics for multiple diseases. Our data suggested that TUG1 is a promising therapeutic target for CS‐induced airway inflammation and fibroblast activation.

## CONFLICT OF INTEREST

The authors confirm that there are no conflict of interest.

## AUTHOR CONTRIBUTIONS

Wenchao Gu and Qiang Li conceived and directed the project; Yaping Yuan and Linxuan Wang analysed the data; Hua Yang, Shanshan Li and Zhijun Tang performed data collection and assembly; Wenchao Gu, Qiang Li and Yaping Yuan designed the experiments, interpreted the data and wrote the paper.
